# The rs10993994 Risk Allele for Prostate Cancer Results in Clinically Relevant Changes in Microseminoprotein-Beta Expression in Tissue and Urine

**DOI:** 10.1371/journal.pone.0013363

**Published:** 2010-10-13

**Authors:** Hayley C. Whitaker, Zsofia Kote-Jarai, Helen Ross-Adams, Anne Y. Warren, Johanna Burge, Anne George, Elizabeth Bancroft, Sameer Jhavar, Daniel Leongamornlert, Malgorzata Tymrakiewicz, Edward Saunders, Elizabeth Page, Anita Mitra, Gillian Mitchell, Geoffrey J. Lindeman, D. Gareth Evans, Ignacio Blanco, Catherine Mercer, Wendy S. Rubinstein, Virginia Clowes, Fiona Douglas, Shirley Hodgson, Lisa Walker, Alan Donaldson, Louise Izatt, Huw Dorkins, Alison Male, Kathy Tucker, Alan Stapleton, Jimmy Lam, Judy Kirk, Hans Lilja, Douglas Easton, The IMPACT Study Steering Committee, The IMPACT Study Collaborators, UK GPCS Collaborators, Colin Cooper, Rosalind Eeles, David E. Neal

**Affiliations:** 1 Uro-Oncology Research Group, CRUK Cambridge Research Institute, Cambridge, United Kingdom; 2 The Institute of Cancer Research, Sutton, Surrey, United Kingdom; 3 Royal Marsden NHS Foundation Trust, Sutton, Surrey, United Kingdom; 4 Department of Pathology, Addenbrookes Hospital, Cambridge, United Kingdom; 5 Peter Maccallum Cancer Centre, Victoria, Australia; 6 The Royal Melbourne Hospital and The Walter and Eliza Hall Institute of Medical Research, Parkville, Victoria, Australia; 7 Genetic Medicine, Manchester Academic Health Sciences Centre, Central Manchester University Hospitals NHS Foundation Trust, Manchester, United Kingdom; 8 Catalonian Institute of Oncology, L'Hospitalet, Barcelona, Spain; 9 Wessex Clinical Genetics Service, The Princess Anne Hospital, Southampton, United Kingdom; 10 NorthShore University Health System, Evanston, Illinois, United States of America; 11 Department of Clinical Genetics, Addenbrookes Hospital, Cambridge, United Kingdom; 12 Institute of Human Genetics, Newcastle-Upon-Tyne, United Kingdom; 13 St George's, University of London, London, United Kingdom; 14 Oxford Regional Genetics Centre, Churchill Hospital, Oxford, United Kingdom; 15 St Michael's Hospital, Bristol, United Kingdom; 16 Guy's Hospital, London, United Kingdom; 17 Kennedy Galton Centre, Northwick Park Hospital, Harrow, Middlesex, United Kingdom; 18 Institute of Child Health, London, United Kingdom; 19 Prince of Wales Hospital, Sydney, New South Wales, Australia; 20 Repatriation General Hospital, Adelaide, Australia; 21 Familial Cancer Service, Westmead Hospital, Westmead, New South Wales, Australia; 22 Departments of Clinical Laboratories, Surgery, Medicine, Memorial Sloan-Kettering Cancer Center, New York, New York, United States of America; 23 Cancer Research UK Genetic Epidemiology Unit, University of Cambridge, Cambridge, United Kingdom; Memorial Sloan-Kettering Cancer Center, United States of America

## Abstract

**Background:**

Microseminoprotein-beta (MSMB) regulates apoptosis and using genome-wide association studies the rs10993994 single nucleotide polymorphism in the *MSMB* promoter has been linked to an increased risk of developing prostate cancer. The promoter location of the risk allele, and its ability to reduce promoter activity, suggested that the rs10993994 risk allele could result in lowered MSMB in benign tissue leading to increased prostate cancer risk.

**Methodology/Principal Findings:**

MSMB expression in benign and malignant prostate tissue was examined using immunohistochemistry and compared with the rs10993994 genotype. Urinary MSMB concentrations were determined by ELISA and correlated with urinary PSA, the presence or absence of cancer, rs10993994 genotype and age of onset. MSMB levels in prostate tissue and urine were greatly reduced with tumourigenesis. Urinary MSMB was better than urinary PSA at differentiating men with prostate cancer at all Gleason grades. The high risk allele was associated with heterogeneity of MSMB staining and loss of MSMB in both tissue and urine in benign prostate.

**Conclusions:**

These data show that some high risk alleles discovered using genome-wide association studies produce phenotypic effects with potential clinical utility. We provide the first link between a low penetrance polymorphism for prostate cancer and a potential test in human tissue and bodily fluids. There is potential to develop tissue and urinary MSMB for a biomarker of prostate cancer risk, diagnosis and disease monitoring.

## Introduction

Microseminoprotein-beta (MSMB) is the second most abundant protein found in semen after prostate-specific antigen (PSA) [1]. Unlike PSA, MSMB is not directly regulated by androgens and its level is not affected by hormone manipulation [2], [3], [4]. Prostate specificity of MSMB expression was initially demonstrated in rodents [5], [6] and supported by the development of a prostate mouse model that used the *msmb* promoter/enhancer region to target prostate specific expression [7], [8]. However studies in humans suggest MSMB can be expressed in a number of secretory and mucosal tissues, albeit at much lower levels than in prostate [9], [10], [11], [12], [13], [14].

Two genome wide association studies (GWAS) have identified a tag small nucleotide polymorphism (SNP), rs10993994, on 10q that is strongly associated with an increased risk of developing prostate cancer [15], [16]. This SNP is in the 5′ UTR of *MSMB*. The risk allele is common, with a frequency of ∼30–40% in Europeans and 70–80% in men of African ancestry, and confers an increased risk of prostate cancer of 1.3 (per allele odds ratio). This association is observed in both Europeans and African-Americans and appears to be independent of age of diagnosis and tumour grade [17]. Fine scale mapping has not identified any other independently associated variants in the region. Furthermore, a recent study in cell lines has shown that the risk allele (T) significantly reduced the promoter activity of the gene to 13% of that in the low risk allele (C) [18]. As this SNP lies within the *MSMB* proximal promoter region reduced promoter activity is suggested to occur via altered transcription factor binding sites such as CREB [19]. These data strongly suggest that the rs10993994 T allele is causally associated with prostate cancer risk, and that this association may be mediated through reduced expression of the MSMB protein in benign prostate tissue. It has been reported that *MSMB* mRNA in cell lines was reduced in cells homozygous for the risk allele, which suggests a hypothesis whereby reduced MSMB expression in individuals with the rs10993994 risk allele could lead to reduced regulation of cell growth and an increased risk of tumourigenesis [19].

Several groups have studied MSMB expression in prostate cancer and all have found higher levels of MSMB in benign and normal tissue or serum when compared with material from tumours [2], [3], [4], [12], [20], [21], [22], [23]. To date no studies have been completed in urine. High levels of MSMB in benign tissue is consistent with the hypothesis that MSMB binds to cell surface receptors and regulates prostate growth by controlling apoptosis possibly via MAP kinase/AKT signalling [24], [25], [26].

Prostate cancer is the most common solid male cancer in the UK. At present the best diagnostic tool is serum PSA while urinary PCA3 mRNA is also used as a useful diagnostic test particularly for men with raised PSA and an initial negative biopsy [27]. However, the need for a digital rectal exam prior to testing precludes its use as a screening tool. Urinary PSA has been reported as a potentially useful diagnostic tool in men with serum PSA 2.5–10 ng/ml and is of particular interest in screening and monitoring disease as it is more acceptable to patients.[28].

We have used immunohistochemical studies of MSMB in benign prostate tissue to demonstrate a relationship between MSMB protein expression and the rs10993994 genotype. We have developed a urinary ELISA assay to examine the association between urinary PSA, MSMB levels and MSMB genotype to determine if this has potential for clinically use as a screening or diagnostic tool.

## Materials and Methods

### Ethics Statement

Full ethical approval was obtained for all human sample collections from either the West Midlands Multi-Centre Research Ethics Committee or the Trent Multi-Centre Research Ethics Committee. All samples were obtained with written consent and analysed anonymously.

### Patient cohorts

Tissue, blood and urine samples from patients with diagnosed prostate cancer were obtained from Addenbrooke's Hospital, Cambridge, UK (radical prostatectomies collected between 2001 and 2008) and The Institute of Cancer Research (biopsy and trans-urethral prostatectomies collected from 1995–2002). Normal/benign patients were recruited as part of the IMPACT study, a study of prostate cancer risk in men with *BRCA1/2* germline mutations at The Institute of Cancer Research (05/MRE07/25) [29]. This cohort had no history of prostate disease and included men with mutated and wild-type (control) *BRCA1* and *BRCA2*. Median age, PSA and Gleason scores of the cohorts used are given in Figure S1.

**Figure 1:**
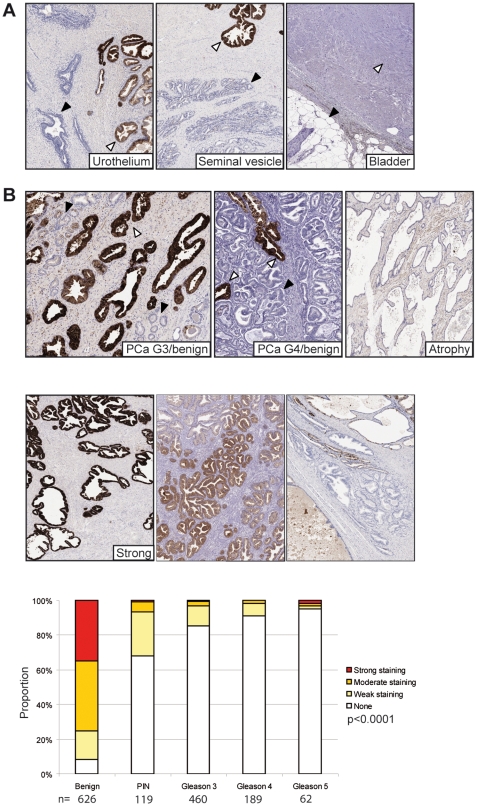
MSMB immunohistochemistry is specific for benign prostate glands. MSMB immunohistochemistry was performed on TMAs using a BondMax autostainer. For all sections nuclei are shown in blue and MSMB staining in brown. (A) MSMB was not present in urothelium (left panel), black arrow - urothelium, white arrow - prostate, seminal vesicle (central panel), black arrow - seminal vesicle, white arrow - prostate, bladder (right panel), black arrow - fat cells, white arrow - bladder muscularis propria. (B) MSMB staining in the prostate is specific for benign glands (white arrows) and not prostate tumour cells (Gleason 3) (black arrows). MSMB also failed to stain atrophic benign glands. Examples of the staining criteria (none/weak, moderate and strong) applied to the immunohistochemistry. Results of the staining were highly significant as calculated using a Kruskal-Wallis test. n represents the number of pathological events scored.

Not all patients had good quality tissue for IHC, DNA for genotyping, serum PSA measurements and urine available. To overcome this, patients were split into three cohorts to answer specific questions. 168 patients with good quality tissue were used for the IHC study depicted in Figure 1. Samples from 133 of the patients were made into a tissue microarray (TMA) containing multiple cores from normal/benign regions (>3), prostatic intra-epithelial neoplasia (PIN) and at least two distinct regions of tumour for each patient. The remaining cases were part of an additional TMA of young onset prostate cancer patients (defined as diagnosed at ≤60 years) from the UK Genetic Prostate Cancer Study. This TMA included multiple normal/benign and tumour regions for 35 patients. n =  indicates the number of events rather than the number of cores examined i.e. where heterogeneous pathology was found both regions were scored independently.

**Figure 2:**
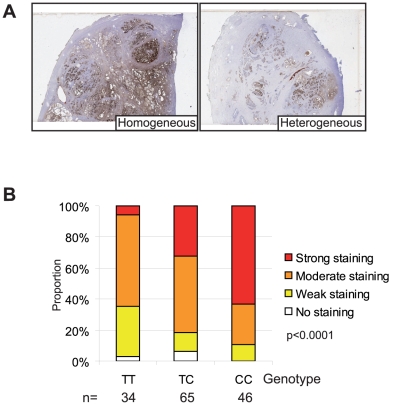
The rs10993994 SNP correlates with MSMB protein expression in benign glands by immunohistochemistry. (A) An example of mega-block staining of large prostate sections showing homogenous (left panel) or heterogeneous (right panel) staining. MSMB immunohistochemistry was scored as before as strong, moderate, weak or lost for each patient and stratified according to genotype; T - high risk, C- low risk. p-values were calculated using a Kruskal-Wallis test. n represents the number of pathological events scored.

Only 145 patients had DNA which could be used for genotyping as shown in Figure 2B. Complete radical prostate sections or very large tissue sections were available from 32 patients from the Addenbrookes cohort.

**Figure 3:**
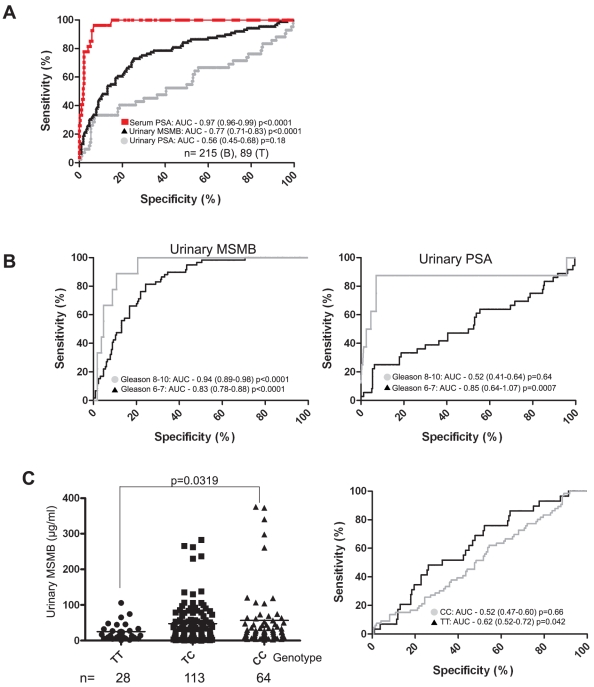
The rs10993994 SNP correlates with urinary MSMB protein expression. Urinary MSMB and PSA concentrations were determined and normalised to creatinine. (A) Urinary MSMB was determined by ELISA for the normal/benign patient cohort and a smaller cohort with a prostate cancer diagnosis. The same cohort was also tested for urinary PSA and serum PSA values collated. ROC curves were generated; AUC  =  area under the curve, confidence intervals are given in brackets and p values are given at 95% confidence levels. The difference between urinary MSMB and PSA ROC curves p = 0.0021. The difference between the urinary MSMB and The tumour cohort was further stratified by Gleason sum score (B). Urinary MSMB and PSA from low Gleason samples (6 and 7) were compared to high Gleason (8 and 9) samples and ROC curves generated and displayed as before. Urinary MSMB was also stratified according to the rs10993994 risk allele (C). n =  the number of individuals examined in each group.

For the MSMB ELISA shown in Figure 3 89 men with prostate cancer that had tissue, DNA and a urine sample were used. The normal/benign patients were recruited as part of the IMPACT study, a study of prostate cancer risk in men with *BRCA1/2* germline mutations (n = 215). All urine samples were obtained prior to any digital rectal exam, aliquoted and frozen immediately at −80°C. Where available age, PSA and Gleason score at diagnosis was determined for each patient.

### Genotyping

Genotyping was completed as previously described [15]. DNA was extracted from whole blood for series 2 and 3, either commercially by GeneProbe or with Qia-amp® DNA Mini Kit (Qiagen) according to manufacturer's instructions. Genotyping of samples was performed by 5′exonuclease assay (Taqman™) using the ABI Prism 7900HT sequence detection system according to the manufacturer's instructions. Primers and probes were supplied directly by Applied Biosystems as Assays-By-Design™.

### Immunohistochemistry

All immunohistochemistry (IHC) was performed using a Bondmax automated stainer and anti-MSMB antibody (Abcam) (1∶400) on tissue from series 1 and 2. For the multi-normal/tumour array a 48 core TMA from Stretton Scientific was used. Nuclei were counterstained with hematoxylin and slides imaged using an Aperio scanning system. Pathology was confirmed by a specialist uro-pathologist (AW) prior to the scoring of any immunohistochemical staining. Scoring was performed by two observers, one of whom was a uro-pathologist (AW), and a consensus obtained. Sequential hematoxylin and eosin stained sections were used to confirm pathology. Staining was graded as; ‘none’ - no staining, ‘weak’ - not all epithelial cells stained and pale staining, ‘moderate’ – most or all epithelial cells stained moderately, ‘strong’ - all epithelial cells stained strongly (Figure 1B). Where sections contained heterogeneous staining an overall consensus was achieved based on the relative proportions of the different scores. Where heterogeneous pathology existed within cores e.g. benign and tumour regions side-by-side, each region was scored as a separate event. n =  indicates the number of events rather than the number of cores examined.

### PSA and MSMB measurements

Serum and urinary PSA (Free & Total) & creatinine assays were performed by the NIHR Cambridge Biomedical Research Centre Core Biochemistry Assay Laboratory, Addenbrookes Hospital using the ProStratus Free/Total PSA DELFIA kit (Perkin Elmer Life & Analytical Sciences) and Dimension RXL analyser (Siemens Healthcare). For the MSMB ELISA all procedures were performed with shaking at room temperature. Wells were washed between each step three times with 0.05% PBS-Tween and a BioTek Elx50 platewasher. Ninety-six well plates (Fisher Scientific) were coated overnight at 4°C in mouse anti-PSP94 antibody (1∶1000, Abcam) diluted in PBS. Wells were blocked with 1% BSA (Sigma) for 1 hour before 50 µl of sample or standard was added to each well in duplicate and incubated for a further 2 hours. Serial dilutions (25−0.78 µg/ul) of recombinant PSP94 (rPSP94, R&D Systems) were used as a control on each plate. Goat anti-MSMB (R&D systems 0.2 µg/ml) was added to each well for 1 hour followed by a1 hour incubation with donkey anti-goat-HRP (Abcam, 1∶20,000). To visualize 100 µl TMB (Sigma) was added to each well for 15 mins. The reaction was stopped by acidifying with 50 µl HCl and absorbance measured at 450 nm using a Lucy II spectrophotometer.

The dynamic range of the ELISA was determined to be 0.01–100 µg/ml although the non-linear range was much greater. Any plate exhibiting >20% variability in the standard curve was deemed to have failed and repeated. Intra- and inter-assay variability was 8.8% and 22% respectively. Effects on protein stability were shown to be <20% after testing by freeze thawing 3 times before assaying or incubating pre-diluted rPSP94 -3 for 4 hours at room temperature prior to assaying. Recovery from different fluids was determined to be <20% by diluting rPRDX-3 in either PBS, 0.1% bovine serum albumin (Sigma) or 10% goat serum (Dako Cytomation). MSMB concentration was determined by interpolation of values from non-linear regression of the sigmoidal standard curve. The concentration of all urine samples was normalised using creatinine measured using a creatinine assay kit (R&D Systems) and the slope of standard curve determined by known standards (20–0.3125 mg/dL) and linear regression. Only samples which could be interpolated from the standard curves without extrapolation were included in the analysis.

### Statistical Methods

All statistics including linear regression and interpolation for the ELISA assay were performed using GraphPad Prism 5. To compare sensitivity and specificity of measurements in the tumour cohort compared to the benign cohort receiver-operator curves (ROC) were performed. A 1-way ANOVA test with a Kruskal-Wallis correction was used to determine if tissue or urinary MSMB levels were significantly different between the benign and tumour cohorts when the tumour cohort was stratified according to Gleason grade or genotype. A Mann Whitney two-tailed student t-test was used for all pairwise comparisons. For all experiments a p-value of 0.05 was considered significant.

## Results

Other than in benign prostate no normal or tumourigenic tissues demonstrated any MSMB staining suggesting that at the protein level in tissue MSMB is highly prostate specific (Figure 1A and Figure S2). In particular the tissues surrounding the prostate e.g. urothelium, seminal vesicles and bladder muscularis propria layer showed no staining whereas adjacent benign prostate glands were heavily stained (Figure 1A).

Consistent with previous reports benign prostate glands showed significantly higher levels of staining compared with tumourigenic regions in our cohort (p<0.0001, Figure 1B and Figure S1) [2], [3], [4], [12], [20], [21], [22], [23]. MSMB staining was also consistently lost in prostatic intra-epithelial neoplasia (PIN) compared to benign tissue (p<0.0001) suggesting that it may be an early, causal, event in tumourigenesis. Staining for MSMB in benign tissue ranged from homogeneous, which was most commonly seen with strong staining (Figure 2A, left panel) and variable, heterogeneous staining frequently seen with weak and moderate staining (Figure 2A, right panel).

MSMB staining in tissue was significantly associated with the rs10993994 genotype (p<0.0001), staining being weakest in men homozygous for the high risk allele (TT) and strongest in CC homozygotes (Figure 2B). There was no evidence of an association between MSMB staining and age or PSA at diagnosis in this malignant series of men (Figure S3).

We determined urinary levels of MSMB in 215 normal/benign men with no history of prostate disease and 89 men with prostate cancer (Figure S4). Consistent with the association in prostate tissue there was a significant decrease in urinary MSMB in men with tumours compared with men with no history of prostate disease (Supporting Document 4A) (p<0.0001). Urinary MSMB significantly improved upon urinary PSA, but not serum PSA, for prostate cancer diagnosis in this cohort (difference between urinary MSMB and PSA ROC curves 0.21, 95% CI: 0.075, 0.34, p = 0.0021) (Figure 3A). When the tumour samples were stratified into low (6 and 7) and high (8 and 9) sum Gleason scores urinary MSMB demonstrated high levels of specificity and sensitivity particularly when compared to urinary PSA (Figure 3B). There was a significant decrease in urinary MSMB with the high risk allele compared (TT) to the low risk allele (CC) (Figure 3C) (p = 0.0319) although this was not reflected in a significant difference in ROC curves. Raised benign urinary MSMB is unlikely to reflect an increased benign prostate volume as there was no significant increase in urinary MSMB with age (Supporting Document 4B) (p = 0.543). To demonstrate that the correlation was specific for the rs10993994 genotype we compared urinary MSMB levels and BRCA1 and BRCA2 germline mutation status, which are known prostate cancer risk factors [30], [31], and found no link (Figure S4).

## Discussion

This is the first study to describe the relationship between the risk allele of the tag SNP rs10993994 and MSMB levels in benign and tumour prostate tissue and to correlate the protein level with genotype and aggressiveness of prostate cancer. The almost complete loss of MSMB in PIN (Figure 1B) suggests that reduced MSMB is an early event in tumourigenesis consistent with an association between prostate cancer risk and the rs10993994 genotype.

There is strong evidence linking the rs10993994 SNP in the *MSMB* promoter region to an increased risk of developing prostate cancer [15], [16]. MSMB has been studied by a large number of groups as a putative prostate cancer biomarker because levels in tissue and serum is reduced or lost with cancer development [2], [3], [4], [12], [20], [21], [22], [23] (Figure 1B). We have confirmed these findings and also that MSMB is largely prostate specific at the protein level in tissue (Figure 1A and Figure S2). Recent evidence suggests that once prostate tumours have developed rs10993994 has little effect on disease progression indicating that any increase in risk affects the benign gland and initial tumour development [17]. This is consistent with the loss of MSMB that we have noted in PIN, which suggests that reduced or lost MSMB expression in benign prostate is required for malignant transformation. The link between the MSMB risk allele and expression in tissue was highly significant (p<0.0001) (Figure 2B). Pronounced heterogeneity was also noted in tissue with moderate to low expression most commonly linked to the TT allele (Figure 2A).

Several groups have examined MSMB levels in serum demonstrating prognostic value after radical prostatectomy [3], [32]. Consistent with the loss of MSMB in tumourigenic glands (Figure 1B) we have demonstrated a highly significant loss of MSMB in urine from patients with both low and high Gleason grade prostate cancer was compared to those with no known prostate disease (Figure 3A). These results compared favourably with urinary PSA which has previously been reported to have potential clinical utility in patients with a serum PSA of 4–10 ng/ml [28]. Urinary MSMB was not as accurate as serum PSA, the current gold standard test for diagnosing prostate cancer (AUC of 0.97 for serum PSA and 0.77 for urinary MSMB. However, our cohorts does not represent the false positive and negatives seen when PSA is used for population screening suggesting a less biased cohort should be tested establish the true benefit of urinary MSMB for prostate cancer screening. Although it is perhaps surprising that such changes are so easily detectable given the potentially small number of tumourigenic glands within an otherwise benign prostate. However, we have shown almost complete prostate specificity for MSMB protein suggesting it is unlikely that significant levels of MSMB arise from any other tissue. It is likely that a proportion of men in our normal/benign study (median age of 53 years) will have some degree of benign prostatic hyperplasia (BPH) but we noted no significant increase in urinary MSMB with increasing age suggesting that increased prostate volume has little effect on urinary MSMB (Figure S4).

Urinary differences in MSMB with the risk allele were significant but not sufficiently discriminatory our relatively small cohort. Changes were independent of other established genetic risk factors such as *BRCA1* and *BRCA2* mutation (Figure S4). Increased assay sensitivity and testing a representative population-based cohort compared to PSA are more likely to demonstrate any true clinical significance in using urinary MSMB to estimate risk and diagnose prostate cancer. Urine is a highly acceptable biological measure for patients and our findings indicate that MSMB is a potential target for further investigation as a urinary biomarker for prostate cancer screening and diagnosis, without the need for a digital rectal exam. Unlike PSA, previous reports have suggested that MSMB levels are unaffected by hormone ablation therapy frequently used in prostate cancer treatment and we need to determine if MSMB might be useful in monitoring disease burden in patients undergoing various forms of treatment [2], [3], [4]. MSMB may also prove useful in detecting those patients with raised PSA but a negative biopsy who would benefit from further investigation.

Quantitative RT-PCR studies of cell lines has previously demonstrated a link between the rs109939994 T allele and lowered MSMB mRNA, most likely due to altered transcription factor binding [19]. The demonstration of a similar association with urinary MSMB and MSMB staining in benign prostate tissue reinforces the causal association between MSMB expression and prostate cancer. Within the prostate MSMB is believed to regulate apoptosis via cell surface receptors, MAP kinase and AKT [24], [25]. We hypothesise that reduced MSMB expression in men with the high risk allele may reduce prostate cell apoptosis and ultimately promote less controlled prostate growth. The loss of such a major regulatory pathway could promote tumourigenesis and provide an explanation for an increased risk of developing prostate cancer while having little effect on outcome once cancer has developed [17].

Our findings raise the possibility that urinary MSMB related to genotype may be a useful biomarker for screening for prostate cancer risk and development. This needs to be studied further to target those men at increased risk for increased surveillance and early intervention and identify those men with prostate cancer.

## Supporting Information

Figure S1 (A) In total 168 tumour patients were studied but not every patient had tissue, DNA and urine available. From the cohort 168 had tissue which included benign which was used in Figure 1. 145 patients had tissue and DNA and were included in Figure 2. 89 patients also had a urine sample and could be used for the ELISA shown in Figure 3. (B) Details of the benign and tumour cohorts used in the study.
(0.73 MB EPS)

Figure S2 MSMB is prostate specific in normal and tumour tissues. A multi-normal tissue TMA was stained for MSMB using immunohostochemistry and a Bondmax autostainer. The array contained multiple cores from the following tissues; skin, breast, thyroid gland, oesophagus, kidney, bladder, lung, colon, liver, ovary, uterus and stomach. Representative images from testes (seminoma tumour), breast and prostate are shown. MSMB staining is brown, nuclei are blue.
(7.19 MB EPS)

Figure S3 The correlation between genotype and age/PSA at diagnosis and immunohistochemical staining and age/PSA at diagnosis. n =  the number of individuals examined in each group. p-values were calculated using a 1-way ANOVA with a Kruskal-Wallis correction.
(1.21 MB EPS)

Figure S4 (A) Urinary MSMB levels were compared by ELISA and a 2-tailed t-test. (B) Urinary MSMB values were analysed according to the age of the individual (years) at the time of sample collection. (C) PSA measurements for the benign cohort stratified according to the rs10993994 genotype. (D) Urinary MSMB does not alter with BRCA mutations. Benign men in Cohort 3 were genotyped for mutations in BRCA 1 and BRCA 2 which are known to confer risk of developing prostate cancer. BRCA genotype was compared with urinary MSMB. Control - no mutation, BRCA 1 - mutation in BRCA 1, BRCA 2 - mutation in BRCA 2. For all graphs n =  number of samples analysed and p-values were calculated using a 1-way ANOVA with a Kruskal-Wallis correction.
(2.03 MB EPS)
